# Supplementary material for: Dispersion patterns of SARS-CoV-2 variants Gamma, Lambda and Mu in Latin America and the Caribbean
Source: Nat Commun. 2024 Feb 28;15:1837. doi: 10.1038/s41467-024-46143-9 (PMC10902334; doi:10.1038/s41467-024-46143-9)
Supplement: Supplementary file 1 — Supplementary Information [file 41467_2024_46143_MOESM1_ESM.pdf]

Supplementary Table 1. Sequencing capacity of Latin American and Caribbean countries under the support of the COVID-19 Genomic Surveillance Regional Network (COVIGEN)

Sequencing Capacity	Country	Laboratory	Acronym
Reference Sequencing Laboratory	Brazil	Fundação Oswaldo Cruz	FIOCRUZ
	Chile	Instituto de Salud Pública de Chile	ISPCH
	Costa Rica	Instituto Costarricense de Investigación y Enseñanza en Nutrición y Salud	INCIENSA
	Mexico	Instituto de Diagnóstico y Referencia Epidemiológicos	InDRE
	Panama	Instituto Conmemorativo Gorgas de Estudios de la Salud	ICGES
	Trinidad and Tobago	University of the West Indies	UWI
	USA	Centers for Disease Control and Prevention	CDC
External sequencing	Bahamas	Reference Laboratory of the Ministry of Health	RL-MoH
	Belize	Medical Laboratory Service of the Ministry of Health and Wellness	MLS-MoHW
	Guyana	National Public Health Reference Laboratory	NPHRL
	Jamaica	University of West Indies	UWI
	Suriname	Centraal Laboratorium Bureau voor Openbare Gezondheidszorg	CL-BOG
	Turks and Caicos	National Public Health Laboratory	NPHL
	Venezuela	Instituto Nacional de Higiene Rafael Rangel	INHRR
In country sequencing	Argentina	Administración Nacional de Laboratorios e Institutos de Salud "Dr. Carlos G. Malbrán"	MALBRAN
	Barbados	Best dos Santos Public Health Laboratory	PHL
	Bolivia	Centro Nacional de Enfermedades Tropicales	CENETROP
	Brazil	Instituto Adolfo Lutz	IAL
	Brazil	Instituto Evandro Chagas	IEC
	Colombia	Instituto Nacional de Salud	INS
	Dominican Republic	Laboratorio de Salud Publica Dr. Defilló	LSPDD
	Ecuador	Instituto Nacional de Investigación en Salud Pública	INSPI
	El Salvador	Laboratorio Nacional de Salud Publica "Dr. Max Bloch"	LNSP
	Guatemala	Laboratorio Nacional de Salud	LNS
	Haiti	Laboratoire National de Santé Publique	LNSP
	Honduras	Laboratorio Nacional de Virología	LNV
	Paraguay	Laboratorio Central de Salud Pública	LCSP
	Peru	Instituto Nacional de Salud	INS
	Uruguay	Departamento Laboratorio de Salud Pública	DLSP

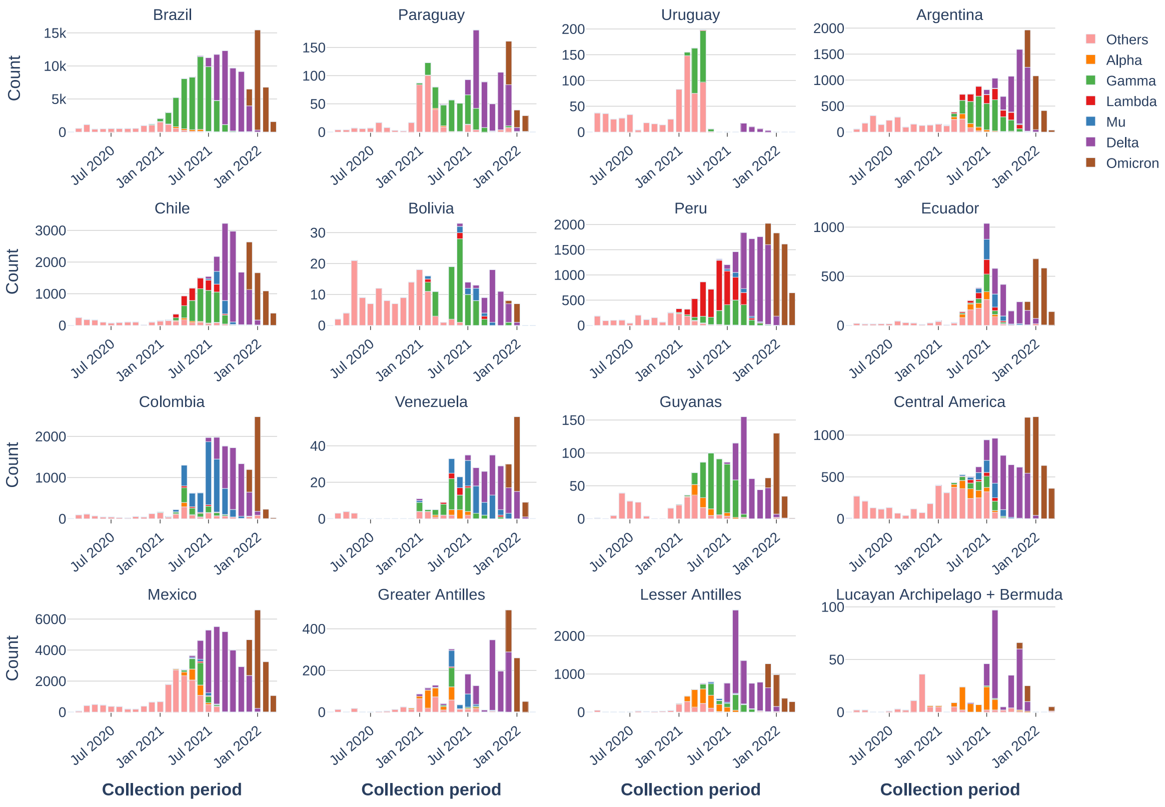

Supplementary Figure 1. Monthly number SARS-CoV-2 genomes sequenced by each location and colored by the main viral variants circulating between February 2020 and March 2022. Source data are provided as a Source Data file.

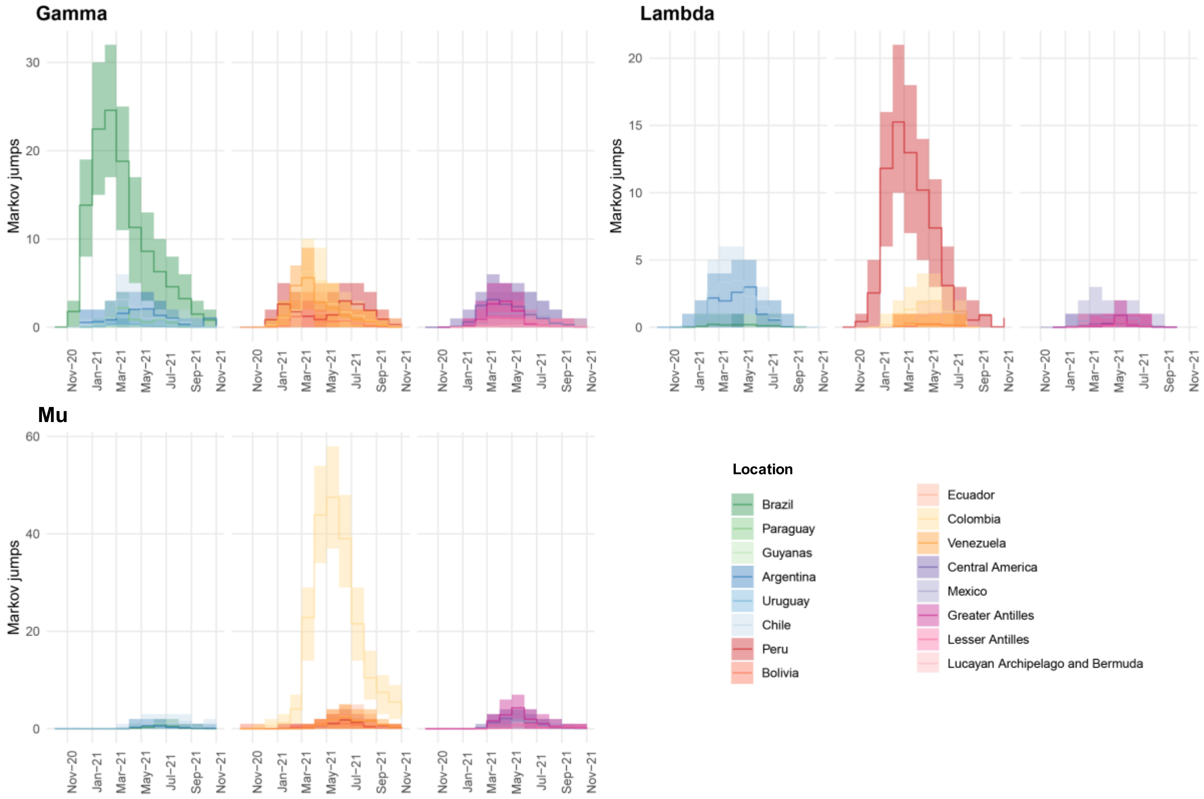

Supplementary Figure 2 –Transitions (Markov jumps) through time from each sampled location for specific SARS-CoV-2 variants. Locations are separated in three groups to facilitate visualization. Source data are provided as a Source Data file.
