# Supplementary material for: Dispersion patterns of SARS-CoV-2 variants Gamma, Lambda and Mu in Latin America and the Caribbean
Source: Nat Commun. 2024 Feb 28;15:1837. doi: 10.1038/s41467-024-46143-9 (PMC10902334; doi:10.1038/s41467-024-46143-9)
Supplement: Supplementary file 3 — Reporting Summary [file 41467_2024_46143_MOESM3_ESM.pdf]

nature portfolio

Corresponding author(s): Tiago Gräf and Juliana Almeida Leite

Last updated by author(s): Jan 29, 2024

Reporting Summary

Nature Portfolio wishes to improve the reproducibility of the work that we publish. This form provides structure for consistency and transparency in reporting. For further information on Nature Portfolio policies, see our Editorial Policies and the Editorial Policy Checklist.

Statistics

For all statistical analyses, confirm that the following items are present in the figure legend, table legend, main text, or Methods section.

n/a	Confirmed	
☐	☒	The exact sample size (*n*) for each experimental group/condition, given as a discrete number and unit of measurement
☒	☐	A statement on whether measurements were taken from distinct samples or whether the same sample was measured repeatedly
☒	☐	The statistical test(s) used AND whether they are one- or two-sided<br>*Only common tests should be described solely by name; describe more complex techniques in the Methods section.*
☐	☒	A description of all covariates tested
☒	☐	A description of any assumptions or corrections, such as tests of normality and adjustment for multiple comparisons
☐	☒	A full description of the statistical parameters including central tendency (e.g. means) or other basic estimates (e.g. regression coefficient) AND variation (e.g. standard deviation) or associated estimates of uncertainty (e.g. confidence intervals)
☒	☐	For null hypothesis testing, the test statistic (e.g. *F*, *t*, *r*) with confidence intervals, effect sizes, degrees of freedom and *P* value noted<br>*Give P values as exact values whenever suitable.*
☐	☒	For Bayesian analysis, information on the choice of priors and Markov chain Monte Carlo settings
☒	☐	For hierarchical and complex designs, identification of the appropriate level for tests and full reporting of outcomes
☒	☐	Estimates of effect sizes (e.g. Cohen's *d*, Pearson's *r*), indicating how they were calculated

Our web collection on statistics for biologists contains articles on many of the points above.

Software and code

Policy information about availability of computer code

Data collection	SARS-CoV-2 genomic data was downloaded from EpiCoV database in GISAID (https://www.gisaid.org/) and air travel data were obtained from the International Air Transport Association (http://www.iata.org). Data processing was performed in R version 4.2.1 (2022-06-23) using RStudio
Data analysis	- R version 4.2.1 (2022-06-23) using RStudio (2023.06.1 Build 524);<br>- IQ-TREE v2.1.2, AliView v.1.27, SeqKit Version: 2.2.0, TempEst v1.5.3;<br>- BEAST v1.10, BEAGLE library v.3, Tracer v1.7,<br><br>Code availability<br>All BEAST xml files used in this study are available at https://github.com/viromol/SC2_LAC-region_phylogeography.git (https://zenodo.org/doi/10.5281/zenodo.10594221)

For manuscripts utilizing custom algorithms or software that are central to the research but not yet described in published literature, software must be made available to editors and reviewers. We strongly encourage code deposition in a community repository (e.g. GitHub). See the Nature Portfolio guidelines for submitting code & software for further information.

Data

Policy information about availability of data

All manuscripts must include a data availability statement. This statement should provide the following information, where applicable:

- Accession codes, unique identifiers, or web links for publicly available datasets
- A description of any restrictions on data availability
- For clinical datasets or third party data, please ensure that the statement adheres to our policy

- Maximum likelihood trees and BEAST xml files are available at https://github.com/akograf/SC2_LAC-region_phylogeography.git (https://zenodo.org/doi/10.5281/zenodo.10594221).
- SARS-CoV-2 genomes used in these analyses were downloaded from EpiCoV database in GISAID (https://www.gisaid.org/) and are available at https://gisaid.org under the EPI_SET_230926ex code locator.
- Proprietary air travel data are commercially available from the International Air Transport Association (https://www.iata.org/) databases and cannot be publicly shared.
- Source data are provided with this paper.

Research involving human participants, their data, or biological material

Policy information about studies with human participants or human data. See also policy information about sex, gender (identity/presentation), and sexual orientation and race, ethnicity and racism.

Reporting on sex and gender	We did not use these variables in our analyses
Reporting on race, ethnicity, or other socially relevant groupings	We did not use these variables in our analyses
Population characteristics	We did not analyze particular characteristic of any human participant. We have only analyzed virus genomes.
Recruitment	Nasopharyngeal positive samples for COVID-19 from routine surveillance were selected to be sequenced based on representativeness and virologic criteria.
Ethics oversight	*Identify the organization(s) that approved the study protocol.*

Note that full information on the approval of the study protocol must also be provided in the manuscript.

Field-specific reporting

Please select the one below that is the best fit for your research. If you are not sure, read the appropriate sections before making your selection.

☒ Life sciences ☐ Behavioural & social sciences ☐ Ecological, evolutionary & environmental sciences

For a reference copy of the document with all sections, see nature.com/documents/nr-reporting-summary-flat.pdf

Life sciences study design

All studies must disclose on these points even when the disclosure is negative.

Sample size	All genomes of Gamma, Lambda and Mu variants sampled in Latin America and Caribbean region with >29,000nt and <5% of Ns were included in the study. To reduce computational burden and sampling disparities among locations, we removed duplicated sequences for each location and subsampled each variant dataset proportionally to the cumulative number of COVID-19 cases attributable to Gamma, Lambda and Mu in each location. An arbitrary number of 1 sequence / 10,000 Gamma attributable COVID-19 cases and 1 sequence / 1,000 Lambda or Mu attributable COVID-19 cases were selected, with a minimum number of 100 sequences (or the maximum available when less than that) per location. To maximize the temporal coverage, sequences were grouped by epidemiological week and sampled as evenly as possible in each location. This approach resulted in datasets of 2,417 Gamma genomes, 1,992 Lambda genomes and 2,618 Mu genomes. These numbers approach the computational limit to Bayesian phylogenetic inferences available for this study and also maintains a good proportion of samples between countries, according to the size of local epidemics. A denser sampling (eg two sequences by 1000 Lambda genomes) could be possible for only a few countries, resulting in the oversampling of the most sequenced locations.
Data exclusions	Genomes with <29,000nt and >5% of Ns and identical sequences sampled in the same location were excluded.
Replication	Not applicable since no experiment was performed.
Randomization	There was no group allocation performed in our study.
Blinding	This study is not a clinical research study and does not involve human subjects thus blinding was not necessary.

Reporting for specific materials, systems and methods

We require information from authors about some types of materials, experimental systems and methods used in many studies. Here, indicate whether each material, system or method listed is relevant to your study. If you are not sure if a list item applies to your research, read the appropriate section before selecting a response.

Materials & experimental systems

n/a	Involved in the study
☒	☐ Antibodies
☒	☐ Eukaryotic cell lines
☒	☐ Palaeontology and archaeology
☒	☐ Animals and other organisms
☒	☐ Clinical data
☒	☐ Dual use research of concern
☒	☐ Plants

Methods

n/a	Involved in the study
☒	☐ ChIP-seq
☒	☐ Flow cytometry
☒	☐ MRI-based neuroimaging
